# Changes in Physical Activity Compared to the Situation before the Outbreak of COVID-19 in Korea

**DOI:** 10.3390/ijerph19010126

**Published:** 2021-12-23

**Authors:** Yoonmi Lee, Seunghui Baek, Jieun Shin

**Affiliations:** 1 Department of Sports Leisure, Sungshin Women’s University, Seoul 02844, Korea; 9988gogo@naver.com; 2 Department of Health Exercise Management, Sungshin Women’s University, Seoul 02844, Korea; 3 Department of Biomedical Informatics, College of Medicine, Konyang University, Daejeon-si 35365, Korea

**Keywords:** Korea community health survey, COVID-19, physical activity

## Abstract

The purpose of this study is to examine changes in physical activity in Korean society, after the outbreak of COVID-19. Method This study was conducted using the Korean Community Health Survey conducted in 2019 and 2020. Subjects that have been diagnosed with hypertension and diabetes were excluded; a total of 355,914 cases were involved for analysis. In terms of the analysis method, General Linear Model (GLM) was conducted to examine the changes in physical activities in 2019 and 2020 depending on the presence of a spouse, educational status, and economic activities. In addition, the GLM was adopted to divide the subjects by gender and age, and analyze their physical activity changes in 2019 and 2020 with spouse presence, educational status, and economic activities as adjusted variables. Result In terms of Koreans, those without a spouse, high educational attainment, and economically inactive were less engaged in physical activities. Differences were found in subjects regarding moderate-intensity physical activities after social distancing following the spread of COVID-19. Senior females without a spouse, both males and females with low educational attainment, economically inactive adult females, and economically active senior males showed a greater drop in physical activities. For walking hours, both adult males and females without a spouse, adult females with all educational attainment level excluding elementary and middle school graduates, and economically inactive adult males and females also showed a downward trend. Conclusion The study recommends that people develop a strategy to increase their post-outbreak physical activity, taking into account the sociodemographic.

## 1. Introduction 

Regular physical activities have proven effective in preventing and managing non-communicable diseases (NCDs) such as heart disease, stroke, diabetes and several types of cancer. However, the World Health Organization (WHO) reported that one in four adults and about 80% of adolescents worldwide lack sufficient physical activity. Physical activity refers to all types of physical movements that cause skeletal muscles to consume energy, such as walking, cycling, sports, recreation and games [1]. As mentioned earlier, physical activities not only pose a positive effect on motor skills, musculoskeletal system, cardiovascular health, and healthy body weight, but are also a critical factor in enhancing general well-being, such as preventing dementia [2,3,4,5,6,7].

As sufficient amount of physical activity can save up to five million lives each year, the WHO recommends that adults older than 18 years old and those 65 years or older perform moderate-intensity physical activities for a minimum of 150 min per week [2]. However, despite the benefits of physical activity, diseases caused by lack of physical activity worldwide are emerging as a social problem; in particular, the rate of aerobic physical activity is decreasing from 62% for men, 55% for women in 2014 to 53% for men, 43% for women in 2019 in Korea [8]. This phenomenon has become more serious due to the COVID-19 virus pandemic [2].

The COVID-19 virus, or SARS-CoV-2 (Severe Acute Respiratory Syndrome Coronavirus 2), appeared worldwide at the end of 2019 [9]. Because of its fast global spread, the WHO declared a global pandemic on 11 March 2020 [10]. More than 100 countries began to execute full or partial lockdown to protect their people from infection and death, controlling the spread through social contact. As quarantine and social distancing began in societies, school classes, workplaces and meetings began to occur online. In addition, as indoor and outdoor public facilities for sports and recreational activities such as the gym, pool and playground closed, all citizens of the world were restricted from engaging in physical activity [11]. Korea was the second country following China to report confirmed cases in the first two months of the outbreak. With the government’s swift public health measures and the active cooperation of the people, the country could reduce the number of new confirmed cases by large, making the world see Korea for responding to the disease without locking it up [12]. However, as the public health policies such as social distancing prolonged in Korea, “Corona Blue,” a newly coined word referring to social depression emerged as well. Corona Blue is a combination of COVID-19 and blues [13]. This may be attributed to reduced physical activity due to prolonged movement restrictions and social distancing, as well as deteriorating mental health such as psychological anxiety, depression, stress, and low mood [14]. 

As such, COVID-19 has greatly transformed the lives of global citizens. While it is suspected that the spread of the pandemic may have reduced people’s physical activity, there is almost no quantitative research on this topic. Hence, this study aimed to examine the engagement of Korean adults (aged 18 or above) and seniors (aged 65 or above) in moderate- and high-intensity physical activities in 2019 and 2020, before and after the outbreak, through the Community Health Survey (CHS). The results of this study will provide basic data for physical activity measures in the upcoming future where the virus has become a part of our daily lives.

## 2. Materials and Methods

### 2.1. Data

This study is a secondary data analysis using raw data from CHS conducted in 2019–2020 (Korea Disease Prevention and Control Agency (kdca.go.kr (accessed on 4 September 2021))). In this study, the 2019–2020 CHS, which was the latest raw data publicly available at the time of data analysis, was used after receiving approval for the use of data for academic research from the Centers for Disease Control and Prevention.

The CHS is conducted every year from August to October on 230,000 adults aged 19 or above nationwide, collecting data about their health status and behaviors in cooperation with 255 public health centers and 35 universities in charge (Source: Community Health Survey, 2019, Korea Centers for Disease Control and Prevention, Cheongju, Korea).

### 2.2. Subjects

Among the subjects (229,099 from 2019, 229,269 from 2020), a total of 355,914 (153,486 from 2019, 202,428 from 2020) were selected for analysis excluding those that have been diagnosed with hypertension and diabetes. Selected subjects from the 2019 result were composed of 44.5% (*n* = 68,265) males and 55.5% (*n* = 85,221) females, and those from the 2020 result were composed of 55.2% (*n* = 111,754) females and 44.8% (*n* = 90,674) males. As for the age, the 2019 survey was composed of 81.3% (*n* = 124,724) adults (19–64 years old) and 18.7% (*n* = 28,762) seniors (65 years and older), and the 2020 survey was composed of 72% adults (*n* = 145,727) and 28% seniors (*n* = 56,701).

### 2.3. Physical Activity

Physical activity was measured through questionnaires [1,2]. The questionnaire used was as follows.

1. In the last week, on how many days to you do involve vigorous-intensity activity, that causes large increases in breathing or heart rate for at least 10 min continuously? How much time do you spend doing vigorous-intensity activities on a typical day?
2. In the last week, on how many days to you do involve moderate-intensity activity, that causes small increases in breathing or heart rate for at least 10 min continuously? How much time do you spend doing moderate-intensity activities on a typical day?
3. In the last week, on how many days to you walk for at least 10 min continuously? How much time do you spend walking on a typical day?

The amount of physical activity was calculated as the total time (minutes) during the week.

### 2.4. Analysis Method

Statistical analysis was performed by IBM SPSS Statistics 25.0. The analysis was conducted to compare the changes in physical activity (high-intensity, moderate-intensity, movement (walking)) surveyed in 2019, before the outbreak, and 2020, after the outbreak. Firstly, the general characteristics of the subjects were described as “*n*” and “%,” and a Chi-square(***χ*^2^**) test was done to compare the two years. In addition, the General Linear Model (GLM) was conducted to examine differences in physical activity in 2019 and 2020 based on spousal presence, educational status and economic activities. Lastly, the GLM was adopted to divide the subjects by gender and age (adults, seniors), and analyze their physical activity changes in 2019 and 2020 with the presence of a spouse, educational status, and economic activities as adjusted variables. All statistical analyzes were tested at the significance level of 0.05.

## 3. Results

### 3.1. General Characteristics of the Subjects before and after the Outbreak of COVID-19

For adult males, the proportion with a spouse decreased from 62.5% to 61.8% after the outbreak. In terms of educational attainment, high school graduates accounted for 42.8% and 43.3% before and after the pandemic, which was the greatest proportion. Economically active adult males declined 2.8%, from 84.4% pre-pandemic to 81.6% post-outbreak. For senior males, the proportion of those with a spouse fell from 88.1% to 86.8% after the pandemic. With respect to educational status, primary school graduates represented the highest proportion of 32.4% and 31.4% pre- and post-outbreak, respectively. Economically active senior males decreased by 3.9% from 52% before to 48.1% after the pandemic. Also, in terms of adult females, the percentage of those with a spouse decreased from 70% before to 68.3% after the outbreak. For educational status, high school graduates accounted for 40.9% before and 41.6% after the pandemic. Adult females engaged in economic activity dropped by 1.3%*p* from 63.3% to 62% after the pandemic. For senior females, those with a spouse fell from 52.7% to 48.5% after the outbreak. In terms of educational status, those with no education took the largest proportion, increasing from 37.5% to 37.9% after the pandemic. Senior females engaged in economic activity fell by 2.8%*p* from 36.5% to 33.7% after the pandemic (Table 1).

### 3.2. Differences in the Amount of Physical Activity Based on General Characteristics before and after the Outbreak of COVID-19

#### 3.2.1. Presence of the Spouse

In regards to high-intensity physical activity, moderate-intensity physical activity, and walking activity, subjects that had their spouse present were more engaged in those activities, except for walking activity of adult females. 

High- and moderate-intensity physical activity in adult males and females revealed a statistically significant difference before and after the COVID-19 outbreak. However, among male and female seniors, only the differences from spousal presence were statistically significant (Table 2 and Table 3). 

On the other hand, for adult males and females, those with a spouse showed a similar increase, but those without a spouse drew a little decrease in walking activity. The interaction between the presence of a spouse and the COVID-19 outbreak seemed statistically significant, whereas the difference before and after the pandemic was also statistically significant in walking activity. However, for senior males and females, only the difference in walking activity based on spousal presence was statistically significant (Table 4).

#### 3.2.2. Educational Status

The high-intensity physical activity, moderate-intensity physical activity, and walking activity of subjects by educational status decreased with higher attainment except for the walking activity of senior females.

Regarding the high-intensity physical activity of males and females in adults and in seniors, only the differences from educational status were found to be statistically significant (Table 2).

For moderate physical activity, adult males and females showed significant differences according to educational status. Such differences were also shown before and after the outbreak of COVID-19 and, the interaction was statistically significant as well. For males, those with higher educational attainment saw a less decrease in the amount of physical activity, while those with graduate school or above education were more engaged in physical activity. For females, those with middle school education increased in physical activity, while those with less educational attainment saw a large drop in physical activity after the outbreak (Table 3). However, senior males and females only showed a statistically significant difference in educational status.

For walking activity, senior males and females as well as adult males were only found to have a significant difference in their educational status, while adult females showed a statistically significant difference in interaction and difference between before and after the pandemic. Subjects with elementary and middle school education increased in walking activity, while other attainment levels dropped in the activity (Table 4).

#### 3.2.3. Engagement in Economic Activity

For high-intensity physical activity depending on the subjects’ engagement in economic activity, adult females showed a statistically significant difference before and after COVID-19 and in whether they are carrying out economic activity. Adult males and senior males and females only showed a statistically significant difference in whether economic activity.

For moderate-intensity physical activity, adult males showed only a statistically significant difference in whether they were economically active, while adult females and senior males appeared to exhibit a statistically significant difference, as well as an interaction between their in whether economic activity and before and after the pandemic. For senior females, there was a statistically significant difference in whether they are engaged in economic activity and before and after the pandemic (Table 2).

For walking activity, both adult males and females were found to have a statistically significant difference in whether economic activity and before and after COVID-19. Also, the interaction was statistically significant. Subjects engaged in economic activity did more walking after the outbreak, while those not engaged in economic activity did less walking after the outbreak. Senior males only showed a statistically significant difference in their engagement in economic activity, while senior females appeared to have a statistically significant difference in their whether economic activity and before and after the outbreak

### 3.3. Differences in Physical Activity before and after COVID-19

The differences in physical activity before and after the outbreak were examined after adjusting the differences shown in the amount of physical activity based on the presence of a spouse, educational status, and economic activity. Also, the actual age of the subjects was additionally adjusted, considering the differences between age groups. For high-intensity physical activity, the amount decreased in all groups after the outbreak, while only adult and senior males showed a statistically significant difference. The amount decreased by approximately 10–20 min in adult and senior males. Meanwhile, moderate-intensity physical activity significantly decreased in all groups. The amount decreased by about 25 min in adult males, 50 min in adult females, 20 min in senior males, and 25 min in senior females. On the other hand, adult males and females did not show a big difference in walking activity, while senior males showed a statistically significant decrease in the activity and senior females had a statistically significant increase in the activity (Table 5).

## 4. Discussion & Conclusions

This study aimed to examine the engagement of Korean adults and seniors in physical activities in 2019 and 2020, before and after the outbreak, through the Community Health Survey (CHS). The results of this study will provide basic data for physical activity measures in the upcoming future where the virus has become a part of our daily lives. After the outbreak of COVID-19, Korea is implementing a policy to prevent the spread of infection through social distancing without national lockdown. It is judged that this will bring about a difference in physical activity change compared to the countries that applied the lockdown policy for the same period. The data from the Community Health Survey (CHS) was used to examine the changes of moderate- and high- intensity physical activities and walk by gender, age, presence of a spouse, educational status, and economic activity. Data were selected for analysis excluding those that have been diagnosed with hypertension and diabetes.

First, time spent on high-intensity physical activity declined among adult and senior males after the COVID-19 outbreak. It was less if they had no spouse, had high education, and were not involved in economic activity. Adult female was significantly reduced the high-intensity physical activity in the status of economic activity before and after social distancing because of COVID-19. According to research conducted in the United Kingdom during the same period which looked at the differences depending on age and gender, young people tended to spend less time than the elderly on high- and moderate- intensity physical activities after social distancing [15]. Also, it reported that differences were found in physical activity by age and gender, as shown in males who were found to be less engaged in physical activities than females [15,16]. In addition, subjects were less likely to carry out physical activities during the lockdown period than during the social distancing period. And as the decline in physical activity was more pronounced in younger people, the study stressed the need for a reactive measure [17]. In addition, there was a marked decrease in high-intensity physical activity in subjects who usually engage in a lot of physical activity. The study suggested that the fall may have been the result of the closure of sports facilities, trails, parks and green spaces [18]. However, in Korea, adults devoted more time to high-intensity physical activity than their older counterparts, and no significant decreases were observed. Physical activity in the Community Health Survey (CHS) identified physical activities at work and recreation (exercise) together, further analysis complementing such aspects needs to be done.

Time spent on moderate-intensity physical activity dropped in all groups after COVID-19. In addition, subjects without a spouse, with high educational attainment and no economic activity were less involved in physical activities. Both adult males and females showed a larger drop with lower educational attainment. On the other hand, this figure showed increase among adult males with a postgraduate degree or higher and among adult females with a middle school diploma. In addition, economically inactive adult females and economically active senior males and senior females without a spouse experienced a significant drop in moderate intensity physical activity. As a result, it is considered that a plan for each subject should be developed to increase their participation in moderate intensity physical activities. While a study reported that the changes in physical activity before and after COVID-19 are not affected by marital status [15], the type of family or cohabitant still needs to be considered as an effective factor to physical activity as the time spent on physical activity is 1.54 times more if one lives with a child than living alone [19]. Especially, efforts must be made to increase physical activity among those living alone or without a partner. Furthermore, low-income people were less physically active than their high-income counterparts after the outbreak [15]. In addition, as the result of observing the physical activity of economically active adults aged 21–40 in Singapore, time spent on walking and moderate-intensity or higher physical activity decreased during periods when movement was limited compared to the initial stage of the outbreak. Moreover, as the trend declined significantly during the lockdown period, it can be seen that social control also plays a role in how much people carry out physical activity [20]. Furthermore, as subjects with low levels of education have experienced significant declines in physical activity, there need to be efforts to increase their participation in physical activity. In addition, further analysis should be carried out considering that educational status is associated with the type of occupation.

Finally, adults did not report changes in walking hours following the COVID-19 outbreak. Senior males showed a slight decrease, while the figure slightly increased in senior females. According to sociodemographic characteristics, little walking time was found in subjects without a spouse excluding adult females, with high educational status in adults, with no educational status in senior and with no economic activity. The walking time between before and after COVID-19 in adult males and females with a spouse increased, and the time for those without a spouse decreased. The walking time for those who were economically active increased and those who were not economically active decreased. In the case of adults who do not have a spouse or are not economically active, it is urgent to encourage physical activity. However, walking time in senior females was found to increase in the presence of a spouse and economic activity before and after COVID-19. In the Community Health Survey, questions about walking include both walking to and from work (or school), walking at exercise and work. After COVID-19, high- and moderate- intensity physical activity decreased significantly in all groups, but walking did not differ in adults. It is judged that all subjects did not participate in the exercise due to the closure of the exercise facility, but maintained physical activity due to work and movement. However, decreasing walking activity in senior males is also predicted to decrease physical activity due to work or movement, it is judged that additional efforts are needed to promote physical activity in senior males. This is a little different result from research conducted in Beijing, China, where daily steps and exercise hours decreased and daily physical activity and sitting time vastly increased, during the forced quarantine periods due to COVID-19 [21]. Also, it is different from the case in Spain, where adults’ walking hours dropped by 58.2% after social distancing and lockdown [18]. The reason for the little difference in walking hours compared to other physical activities in Korea can be found in the situation specific to the country where it is controlling the spread with only social distancing, not locking down the country, which therefore has not greatly affected physical activity in daily life. In addition, the reason for the increase of senior females’ walking activity may have been driven by the shutdown of sports facilities and public spaces due to phased social distancing, which reduced their engagement in moderate-intensity and high-intensity physical activity and encouraged them to move more in daily life as compensation.

As a result, after social distancing due to COVID 19, high- and moderate - intensity physical activities for all subjects decreased in Koreans. The pattern of changes in physical activities was different according to their spouse, education level, and economic activity. Subjects showed a difference in moderate-intensity physical activity after the pandemic-driven social distancing, and the drop was bigger for senior females without a spouse, adult males and females with a low educational level, economically inactive adult females, and economically active senior males. For walking activity, adult males and females without a spouse, adult females with educational level excluding elementary and middle school graduates, and economically inactive adult males and females were found to walk less after the outbreak of COVID-19. Therefore, it is recommended that strategies be put in place to encourage people to have more physical activity, which has decreased after the outbreak, taking into account these sociodemographic factors.

The limitation of this study is that changes in physical activity before and after COVID-19 by individuals were not identifiable as it was based on secondary data. And only general characteristics commonly researched before and after the outbreak were considered. In addition, working time and leisure time could not be separated when measuring time spent on high and moderate intensity physical activities, and subjects with physical disabilities could not be entirely excluded as those with chronic diseases other than hypertension and diabetes could not be ruled out. However, the results of this study may be used as a basis for developing physical activity strategies for the next period of “living with COVID-19” by analyzing the differences in the physical activity carried out by Koreans before and after the outbreak by gender and age.

## Figures and Tables

**Table 1:** General characteristics of subjects.

			Adults			Seniors		
			2019	2020	*χ*^2^(*p*)	2019	2020	*χ*^2^(*p*)
Males	Spouse	Present	34,596(62.5)	41,568(61.8)	5.982(0.015)	11,283(88.1)	20,243(86.8)	11.881(0.001)
		Absent	20,803(37.6)	25,729(38.2)		1529(11.9)	3078(13.2)	
	Educational Status	No education	262(0.5)	323(0.5)	37.347(<0.001)	1602(12.5)	2442(10.5)	56.006(<0.001)
		Elementary	1614(2.9)	2136(3.2)		4149(32.4)	7305(31.4)	
		Middle	3260(5.9)	4302(6.4)		2651(20.7)	4854(20.8)	
		High	23,721(42.8)	29,097(43.3)		2864(22.4)	5780(24.8)	
		College	7969(14.4)	9177(13.6)		229(1.8)	410(1.8)	
		University	15,832(28.6)	18,831(28)		1006(7.9)	1932(8.3)	
		Graduate school or above	2746(5)	3404(5.1)		307(2.4)	574(2.5)	
	Economic Activity	Yes	46,783(84.4)	54,941(81.6)	167.033(<0.001)	6663(52)	11,219(48.1)	50.26(<0.001)
		No	8648(15.6)	12,384(18.4)		6153(48)	12,109(51.9)	
Females	Spouse	Yes	48,433(70)	53,504(68.3)	50.376(<0.001)	8394(52.7)	16,192(48.5)	74.957(<0.001)
		No	20,754(30)	24,842(31.7)		7531(47.3)	17,166(51.5)	
	Educational Status	No education	566(0.8)	761(1.0)	161.067(<0.001)	5974(37.5)	12,645(37.9)	18.814(0.094)
		Elementary	3673(5.3)	4819(6.2)		5636(35.4)	11,905(35.7)	
		Middle	5663(8.2)	7131(9.1)		1973(12.4)	4226(12.7)	
		High	28,285(40.9)	32,594(41.6)		1725(10.8)	3408(10.2)	
		College	10,786(15.6)	11,360(14.5)		155(1)	294(0.9)	
		University	17,786(25.7)	18,930(24.2)		387(2.4)	727(2.2)	
		Graduate school or above	2473(3.6)	2704(3.5)		69(0.4)	120(0.4)	
	Economic Activity	Active	43,813(63.3)	48,563(62)	26.63(<0.001)	5815(36.5)	11,240(33.7)	37.043(<0.001)
		Inactive	25,443(36.7)	29,815(38)		10,117(63.5)	22,103(66.3)	

**Table 2:** Differences in physical activity (high-intensity) by general characteristics of subjects (2019–2020).

			Adults					Seniors				
			2019	2020	*F*(*p*) ^1)^	*F*(*p*) ^2)^	*F*(*p*) ^3)^	2019	2020	*F*(*p*) ^1)^	*F*(*p*) ^2)^	*F*(*p*) ^3)^
Males	Spouse	Present	433.54 ± 637.94	427.95 ± 621.54	4.500	83.270	1.330	595.31 ± 742.56	556.54 ± 687.13	1.320	4.190	0.000
		Absent	387.53 ± 512.88	368.63 ± 510.01	(0.034)	(<0.001)	(0.249)	526.55 ± 623.64	488.64 ± 571.65	(0.251)	(<0.001)	(0.990)
	Educational Status	No education	824.85 ± 1004.85	769.08 ± 1022.23	1.59	376.94	0.26	728.18 ± 908.65	748.6 ± 787.53	2.33	25.24	0.88
		Elementary	894.13 ± 1022.97	874.87 ± 977.78	(0.208)	(<0.001)	(0.955)	712.51 ± 841.62	628.14 ± 787.89	(0.126)	(<0.001)	(0.509)
		Middle	746.42 ± 878.24	709.87 ± 885.51				612.02 ± 812.04	607.22 ± 750.49			
		High	479.36 ± 650.42	468.15 ± 642.39				483.74 ± 546.81	491.09 ± 562.75			
		College	394.57 ± 558.15	379.47 ± 522.38				602.55 ± 665.92	487.96 ± 698.77			
		University	296.96 ± 407.74	286.3 ± 391.81				398.24 ± 376.11	342.26 ± 300.52			
		Graduate school or above	239.17 ± 265.68	241.16 ± 282.12				390.86 ± 473.64	329.65 ± 332.11			
	Economic Activity	Yes	433.5 ± 623.96	423.9 ± 616.73	0.470	252.160	0.280	686.29 ± 845.93	636.19 ± 782.32	2.640	182.130	0.940
		No	304.04 ± 315.07	302.79 ± 314.95	(0.491)	(<0.001)	(0.597)	407.23 ± 389.3	394.6 ± 380.82	(0.104)	(<0.001)	(0.332)
Females	Spouse	Yes	347.18 ± 531.6	324.65 ± 516.7	11.990	195.110	0.130	565.97 ± 741.11	537.83 ± 714	3.370	9.370	0.340
		No	262.63 ± 397.85	244.29 ± 359.54	(0.001)	(<0.001)	(0.723)	510.38 ± 695.47	456.2 ± 651.08	(0.066)	(<0.001)	(0.561)
	Educational Status	No education	797 ± 855.46	812.76 ± 1010.55	3.69	391.09	0.45	636.97 ± 803.72	542.23 ± 707.42	0.21	12.63	1.19
		Elementary	717.28 ± 906.6	669.25 ± 885.51	(0.055)	(<0.001)	(0.842)	620.93 ± 798.08	544.11 ± 756.41	(0.647)	(<0.001)	(0.310)
		Middle	605.45 ± 826.44	562.48 ± 750.77				461.76 ± 639.83	504.31 ± 696.79			
		High	344.99 ± 503.48	327.35 ± 487.19				341.32 ± 411.92	353.95 ± 431.96			
		College	255.84 ± 363.64	230.78 ± 327.91				321.74 ± 399.91	400.8 ± 587.82			
		University	223.39 ± 299.74	194.39 ± 259.09				298.19 ± 274.73	318.49 ± 289.28			
		Graduate school or above	192.48 ± 247.17	173.77 ± 196.49				227.78 ± 253.46	427.5 ± 502.18			
	Economic Activity	Active	355.68 ± 569.33	325.21 ± 529.76	15.140	250.280	1.760	718.38 ± 847.11	651.77 ± 826.52	3.380	275.390	1.710
		Inactive	255.58 ± 301.68	240.62 ± 312.66	(<0.001)	(<0.001)	(0.184)	339.27 ± 467.4	328.04 ± 411.12	(0.066)	(<0.001)	(0.191)

^1)^ Before and after the outbreak (2019–2020); ^2)^ Differences in general characteristics; ^3)^ Interaction between general characteristics and before and after COVID-19.

**Table 3:** Differences in physical activity (moderate-intensity) by general characteristics of subjects (2019–2020).

			Adults					Seniors				
			2019	2020	*F*(*p*) ^1)^	*F*(*p*) ^2)^	*F*(*p*) ^3)^	2019	2020	*F*(*p*) ^1)^	*F*(*p*) ^2)^	*F*(*p*) ^3)^
Males	Spouse	Present	505.28 ± 698.54	491.27 ± 689.12	15.010	155.430	2.320	355.17 ± 404.69	361.02 ± 388.35	0.270	10.450	0.050
		Absent	440.03 ± 604.73	407.84 ± 575.99	(<0.001)	(<0.001)	(0.128)	331.78 ± 413.49	334.02 ± 370.85	(0.604)	(0.001)	(0.818)
	Educational Status	No education	1015.85 ± 1105.79	696.33 ± 815.01	14.05	423.35	4.11	734.59 ± 779.29	688.11 ± 747.04	3.89	35.13	2.09
		Elementary	933.02 ± 1006.85	840.98 ± 954.65	(<0.001)	(<0.001)	(<0.001)	768.08 ± 846.23	654.97 ± 744.9	(0.049)	(<0.001)	(0.052)
		Middle	812.54 ± 905.88	733.51 ± 884.25				689.7 ± 827.93	601.65 ± 694.63			
		High	552.81 ± 712.79	526.5 ± 702.32				540.12 ± 629.81	532.29 ± 653.27			
		College	472.01 ± 647.06	439.02 ± 597.26				538.74 ± 786.77	504.08 ± 636.9			
		University	333.85 ± 488.34	330.19 ± 497.58				421.75 ± 505.76	438.2 ± 514.46			
		Graduate school or above	245.9 ± 310.44	252.77 ± 330.81				434.84 ± 546.05	417.15 ± 475.02			
	Economic Activity	Yes	509.39 ± 697.83	492.6 ± 688.77	2.720	680.280	0.140	774.87 ± 864.12	694.49 ± 780.06	12.750	577.760	4.560
		No	289.39 ± 328.44	278.73 ± 315.05	(0.099)	(<0.001)	(0.712)	406.2 ± 417	385.99 ± 407.64	(<0.001)	(<0.001)	(0.033)
Females	Spouse	Present	452.67 ± 671.91	439.33 ± 661.59	8.040	428.920	0.430	586 ± 697.76	589.89 ± 720.18	2.970	57.340	4.120
		Absent	329.96 ± 513.77	308.6 ± 498.58	(0.005)	(<0.001)	(0.512)	515.16 ± 648.92	467.2 ± 576.7	(0.085)	(<0.001)	(0.043)
	Educational Status	No education	864.81 ± 957.16	793.31 ± 914.88	6.75	587.24	4.18	609.02 ± 698.53	533.59 ± 643.8	0.51	28.21	1.31
		Elementary	845.8 ± 939.34	770.31 ± 853.71	(0.009)	(<0.001)	(<0.001)	616.47 ± 731.67	608.29 ± 724.87	(0.476)	(<0.001)	(0.248)
		Middle	664.31 ± 814.43	718.55 ± 894.43				504.63 ± 615.66	504.48 ± 648.73			
		High	459.11 ± 672.35	422.88 ± 631.36				398.35 ± 542.24	397.51 ± 533.22			
		College	330.03 ± 507.05	312.99 ± 503.71				349.8 ± 505.99	396.58 ± 522.84			
		University	275.21 ± 431.55	244.55 ± 388.18				312.75 ± 323.29	326.05 ± 450.88			
		Graduate school or above	227.87 ± 336.06	205.81 ± 298.9				465.71 ± 559.21	322.86 ± 221.23			
	Economic Activity	Active	480.19 ± 713.99	467.96 ± 702.2	17.000	1085.640	4.130	757.35 ± 808.3	704.95 ± 785.25	7.870	1024.610	2.350
		Inactive	299.38 ± 416.43	263.38 ± 372.31	(<0.001)	(<0.001)	(0.042)	352.33 ± 425.71	336.98 ± 400.49	(0.005)	(<0.001)	(0.125)

^1)^ Before and after the outbreak (2019–2020); ^2)^ Differences in general characteristics; ^3)^ Interaction between general characteristics and before and after COVID-19.

**Table 4:** Differences in physical activity (walking) by general characteristics of subjects (2019–2020).

			Adults					Seniors				
			2019	2020	*F*(*p*) ^1)^	*F*(*p*) ^2)^	*F*(*p*) ^3)^	2019	2020	*F*(*p*) ^1)^	*F*(*p*) ^2)^	*F*(*p*) ^3)^
Males	Spouse	Present	380.17 ± 516.32	385.62 ± 513.99	1.030	10.480	6.830	355.17 ± 404.69	361.02 ± 388.35	0.270	10.450	0.050
		Absent	378.04 ± 510.04	365.69 ± 513.93	(0.311)	(0.001)	(0.009)	331.78 ± 413.49	334.02 ± 370.85	(0.604)	(0.001)	(0.818)
	Educational Status	No education	452.73 ± 615.55	414.52 ± 497.77	0.00	149.02	1.53	340.88 ± 433.88	325.39 ± 397.12	0.55	2.74	1.23
		Elementary	452.66 ± 572.41	472.63 ± 613.88	(0.980)	(<0.001)	(0.164)	353.95 ± 436.95	353.06 ± 397.37	(0.458)	(0.012)	(0.286)
		Middle	427.74 ± 578.79	455.66 ± 628.5				357.04 ± 411.7	347.73 ± 398.5			
		High	416.83 ± 563.59	406.41 ± 561.22				353.13 ± 384.97	374.41 ± 383.93			
		Community College	395.8 ± 550.33	398.44 ± 557.21				399.2 ± 425.63	398.24 ± 370.24			
		University	323.17 ± 418.8	322.93 ± 399.55				344.53 ± 331.36	359.53 ± 329.97			
		Graduate school or above	276.89 ± 310.76	273.68 ± 297.39				336.7 ± 276.15	368.2 ± 333.65			
	Economic Activity	Yes	394.96 ± 543.06	401.62 ± 547.81	5.360	649.600	14.780	386.34 ± 475.65	388.86 ± 457.15	1.800	161.560	0.710
		No	300.64 ± 317.84	273.79 ± 306	(0.021)	(<0.001)	(<0.001)	317.74 ± 314.61	328.81 ± 304.4	(0.180)	(<0.001)	(0.398)
Females	Spouse	Present	284.55 ± 372.55	292.07 ± 388.5	13.970	10.440	47.150	276.86 ± 307.31	290.52 ± 316.49	6.490	176.260	2.160
		Absent	308.83 ± 404.84	283.33 ± 398.11	(0.000)	(0.001)	(0.000)	236.75 ± 275.81	240.41 ± 267.92	(0.011)	(<0.001)	(0.141)
	Educational Status	No education	339.65 ± 457.59	309.02 ± 409.1	0.91	128.09	3.04	223.19 ± 276.86	231.75 ± 289.9	2.01	38.79	0.11
		Elementary	311.57 ± 408.46	326.68 ± 429.32	(0.341)	(<0.001)	(0.006)	265.37 ± 306.91	272.07 ± 303.55	(0.157)	(<0.001)	(0.995)
		Middle	323.38 ± 427.27	339.79 ± 425.62				287.67 ± 281.8	291.78 ± 289.81			
		High	315.77 ± 415.3	312.75 ± 430.37				291.57 ± 318.04	300.06 ± 297.51			
		Community College	277.85 ± 373.02	263.05 ± 379.02				257.93 ± 217.86	280.98 ± 258.04			
		University	258.11 ± 322.79	246.39 ± 311.46				277.64 ± 270.72	285.76 ± 226.63			
		Graduate school or above	240.23 ± 265.49	238.52 ± 269.16				250.7 ± 212.06	279.48 ± 190.14			
	Economic Activity	Active	311.67 ± 438.05	317.09 ± 452.41	5.390	732.350	21.910	288.43 ± 362.53	298.71 ± 363.92	5.910	188.060	0.230
		Inactive	260.22 ± 266.96	244.13 ± 259.22	(0.020)	(<0.001)	(<0.001)	241.76 ± 244.62	248.64 ± 248.67	(0.015)	(<0.001)	(0.630)

^1)^ Before and after the outbreak (2019–2020); ^2)^ Differences in general characteristics; ^3)^ Interaction between general characteristics and before and after COVID-19.

**Table 5:** Differences in physical activity (minutes spent weekly on physical activity) before and after COVID-19 (2019–2020).

		Adults			Seniors		
		2019	2020	*F(p)*	2019	2020	*F(p)*
Male	high	477.75 ^†^	465.22		407.87	384.29	
		415.45 ± 592.42	404.22 ± 580.21	5.22(0.022) ^††^	320.96 ± 495.74	296.32 ± 468.9	19.56(<0.001)
	mid	489.30	465.90		455.76	431.19	
		481.46 ± 666.63	459.99 ± 650.23	17.58(<0.001)	418.16 ± 633.87	398.95 ± 618.89	21.18(<0.001)
	walk	350.97	351.75		286.98	281.35	
		379.36 ± 514.03	377.67 ± 513.66	0.06(0.814)	292.15 ± 383.18	289.19 ± 391.7	6.37(0.012)
Female	high	510.83	480.74		452.83	415.64	
		589.86 ± 733.74	550.31 ± 678.09	2.71(0.100)	545.59 ± 724.92	506.33 ± 691.34	3.12(0.077)
	mid	539.79	487.11		466.15	439.45	
		650.16 ± 763.94	584.1 ± 687.2	15.53(<0.001)	557.78 ± 677.17	537.18 ± 664.92	4.93(0.026)
	walk	350.70	358.24		259.51	272.92	
		352.52 ± 405.86	357.33 ± 386.02	2.23(0.135)	258.91 ± 294.46	265.88 ± 294.71	15.84(<0.001)

^†^ Least square mean. ^††^ Covariated by actual age, presence of spouse, educational status, engagement in economic activity.

## Data Availability

Data used in this study are available on the website Community Health Survey for Korea Disease Control and Prevention Agency at “https://chs.kdca.go.kr/chs/main.do” (accessed on 4 September 2021).
